# Development, validation and reliability testing of the hospice care environment scale

**DOI:** 10.1186/s12904-024-01450-2

**Published:** 2024-05-28

**Authors:** Junping Zhong, Wei Zhang, Rong Xu, Huifen Wang, Jing Zhao, Yingjuan Huang, Yanlin Chen, Xiaoli Chen, Jianfei Chen, Qing Zhang, Zhijie Zou, Yingzi Zhang

**Affiliations:** 1 School of Nursing, Wuhan University, Located on No. 115 Donghu Road, Wuhan, Hubei province 430071 China; 2 Minxi Vocational & Technical College, Located on No.8 Caoxi Road, Longyan, Fujian province 364021 China; 3 Department of Nursing, Hubei Cancer Hospital, Located on No. 116 Zhuodaoquan South Road, Hongshan District, Wuhan, Hubei province 430079 China; 4 Sir Run Run Shaw Hospital, Affiliated hospital of medical school of Zhejiang University, Hangzhou, Zhejiang province China; 5 School of Medicine, Nankai University, Located on No. 94 Weijin Road, Tianjin, 300071 China; 6 Magnet Program & Research Department, UT Southwestern Medical Center, 8200 Brookriver Dr., North Tower, 5th floor, Dallas, TX 75247 USA; 7 Department of Obstetrics, Guoyang County People’s Hospital, No. 112, Shengli Road, Chengguan Town, Bozhou, Anhui Province 233600 China

**Keywords:** Hospice care, Scale development, Psychometric testing, Validation, Delphi

## Abstract

**Background:**

WHO stated the environment is an important factor affecting the development of hospice care. The environment is the sum of factors affecting behavior besides the individual factors. Currently, a scale to comprehensively assess the hospice environment of nurse is still lacking. This study aimed to develop an instrument to investigate the environmental factors affecting hospice care of nurses.

**Methods:**

Literature review and a semi-structured interview were conducted to form the items pool of the Hospice Care Environment Scale. Two rounds of Delphi expert consultation were conducted by 16 experts to revise the scale dimensions and entries to form the Hospice Care Environment Scale. A psychometric evaluation was then performed among 530 oncology nurses in a large tertiary oncology hospital in Hubei Province. The 500 valid questionnaires were randomly divided into two groups in a 1:1 ratio, sample 1 (n1 = 250) for item screening and sample 2 (n2 = 250) for quality evaluation of the resulting scale. Item analysis, reliability analysis, validity analysis and acceptability analysis were performed.

**Result:**

The Hospice Care Environment Scale consists of two dimensions and 13 entries. The Cronbach’s α coefficient of the Hospice Care Environment Scale was 0.970, and the Cronbach’s α coefficient of the two dimensions were 0.952 and 0.969, respectively, with the Item-content validity index and average Scale- content validity index of the scale was both 1.000. The validation factor analysis showed the standardized path coefficients of each item were basically above 0.5, and the factor structure model was stable and suitable. The average completion time of the scale was about 3 min, which had good feasibility.

**Conclusion:**

The Hospice Care Environment Scale to assess the environment of hospice care services, has good content and construct validity and reliability. This scale can provide guidance to evaluate the hospice care environment.

**Supplementary Information:**

The online version contains supplementary material available at 10.1186/s12904-024-01450-2.

## Background

Hospice care is a system of care delivery for patients at the end of life, including pain and various symptom management, comfort care, and psychological, spiritual and social support [1]. Hospice care is required for most diseases, including cardiovascular diseases (38.5%), cancer (34%), chronic respiratory diseases (10.3%), AIDS (5.7%) and diabetes (4.6%) [2]. It is estimated that only 14% of people who need hospice care worldwide receive it [3]. Further, 80% of terminal patients in low- and middle-income countries lack access to hospice care [4]. Therefore, the providing of hospice care is imminent.

The providing of hospice care is influenced by a variety of factors. Environment is undoubtedly an important factor influencing hospice care, as stated by the World Health Organization [2]. The theory of social cognition developed by Bandura holds that the environment is the sum of factors that influence individual behavior in addition to individual factors [5]. Therefore, there may be many environmental factors that influence the implementation of hospice care. The socio-cultural environment is one of the important factors. For example, traditional beliefs about death and dying can make it difficult for hospice care to be well accepted by the population in eastern countries [6]. In addition, the public have misconceptions about hospice care, such as the perception that it is a way to indulge patients waiting to die or that only cancer patients need hospice care [7, 8]. Also, people may misunderstand that increasing access to opioid analgesics will lead to the increased drug abuse [9]. The government also plays an important role in the hospice care environment. Management and policy makers are not sufficiently aware of the benefits and importance of hospice care [10]. In addition, some areas lack the resources needed to train and deliver palliative care [6].

China, as the largest developing country, faces the same dilemma. In response to the increasing demand for hospice care, China has been devoting great efforts to improve its environment. Since 2015, China has released and implemented a series of actions to improve hospice care environment [11]. Through continuous efforts, Chinese hospice care quality improved from the 71st to 53rd place in 2021 [12]. But there is still a considerable gap between China and the world’s advanced level [12]. Therefore, investigation of the hospice care environment is essential. Current measures for evaluating hospice care performance frequently overlook environmental aspects, such as social and organizational dimensions. In addition, previous research has focuses on the hospice care experiences of patients and their families, with little emphasis on medical staff’s perspective on the environment.

Nurses are the major practitioners of hospice care and it is essential to assess their perceptions of hospice care environment. To our knowledge, no scales have been developed to comprehensively assess environment of hospice care. This study aimed to develop, validate and reliability-test an instrument to investigate the hospice care environment of nurses. We hope this study provides new perspectives for assessing hospice care.

## Methods

### Phase I: development of the items pool

This study strictly followed the principles and process recommended by Robert F. DeWillis for the development of the Hospice Care Environment Scale [13]. This principle is widely used in the development of scales [14, 15], consisting of eight steps: clearly identifying what is being measured, establishing a pool of scale entries, determining how the scale should be presented, expert review of the pool of entries, consideration of including test entries, selection of samples for entry testing, evaluation of entries, and optimization of scale length. We first conducted a literature review in multiple scientific databases including PubMed, Web of science, Cochrane Library, PsycINFO and Embase, combined with keywords such as “nursing”, “hospice care”, “environment” and “scale” to gain a comprehensive understanding of hospice care environment, and then relevant items were extracted and defined in the entry pool [13]. Based on the literature analyses, we drawn on assessment tools related to the environment and similar concepts, such as the Practice environment scale of the nursing work index (PES-NWI) [16], the Perceived Nursing Work Environment scale (PNWE) [17]. In this study, we assessed hospice care conducted within institutions. Therefore, this scale assesses nurses working in institutions such as hospitals and hospice wards in a hospice setting.

We then conducted semi-structured interviews, including 12 oncology nurses from four tertiary hospitals in Wuhan, Hubei Province from March to May 2021, to collect their perception about relevant environment indicators and to suggest possible indicators to be included in an instrument. These semi-structured interviews were recorded and transcribed verbatim to identify possible themes, topics, and candidate indicators. Nurses consented to voluntarily participate and their anonymity was assured throughout the process. The semi-structured interviews are as follows.

- What do you think about hospice care? Why?
- What factors influence you to practice hospice?
- What environmental factors (other than your own psychological and cognitive factors) affect your hospice practice?
- How do different environmental factors affect your hospice care?
- Do you have any additional questions about this interview (environmental factors in your hospice practice)?

The research team synthesized the collected information and formed an initial pool of entries. During a one-day workshop, the research team revised semantically unclear or lengthy expressions, and merged similar entries and then, by consensus, proposed a final hospice care environment scale entry pool that were able to assess all the areas that were identified in the literature process or in the semi-structured interviews.

### Phase II: selection of environment indicators through consensus process

To assess content validity, the proposed environment indicators were presented to a panel of experts from different disciplines, with five or more years of clinical experience in the field. Through a consensus process, the experts were asked to identify the indicators that were relevant to the hospice care environment and that were important. This consensus process was performed using an on-line questionnaire with a two-round modified Delphi’s methodology designed in accordance with the Delphi Method implementation steps and procedures [18]. In the first round, and after consenting to participate, experts were informed about the purpose of the study, the background, the evaluation content, and were asked to rate the relevance and importance of each indicator on a scale of 1–4. Higher scores represented higher relevance and importance. Experts had one week to complete the survey. After recovering the first round of correspondence questionnaires, the research team collected and analyzed the experts’ opinions, and after thorough discussions, adjusted and modified the dimensions and items of the scale. After forming a new expert consultation questionnaire, a second round of expert consultation was conducted. The interval between the two rounds of expert consultation is at least 4 weeks. If there is a convergence of expert opinion, the correspondence would be ended. Two rounds of correspondence were eventually conducted for this study, and the correspondence period was from May to August 2021.

### Phase III: psychometric evaluation of the hospice care environment scale

#### Participants and procedure

All oncology nurses were recruited from two tertiary care hospitals in Wuhan China. Recruitment took place between October and November 2021, who met the following inclusion criteria: (a) age over 18 years old; (b) registered clinical nurses with nurse qualification certificate; (c) working in the oncology department, and working years ≥ 1 year; (d) on-the-job work during the investigation. The exclusion criteria: (a) non-survey hospital nurses in the survey hospital for further study, practice, and training; (b) currently not engaged in clinical work. Ethical approval was obtained from the Medical Faculty of Wuhan University (No.2020YF2001).

#### Measures

A pilot test was taken on 25 oncology nurses from 2 hospitals in Wuhan. The nurses were asked to complete the scale and provide written comments and suggestions about the scale in terms of the format, content, comprehensibility, and ease of reading.

From October to November 2021, a formal questionnaire survey was conducted using the Questionnaire Star platform. After obtaining permission from the hospital, the researchers sent questionnaires to all head nurses in the oncology department, detailing the purpose of the questionnaire, inclusion and exclusion criteria, etc. The head nurses sent the questionnaire link to the department working group, explaining the purpose and significance of this study, the rights and their contact information of the study subjects, and set “I agree to participate” and “I don’t agree to participate” options. If the subject selects the “I agree to participate” option, the page will jump to the formal questionnaire. All questions in the questionnaire were set as mandatory, and the subjects could submit the questionnaire only after filling in all questions. A total of 530 oncology nurses from the two hospitals completed the questionnaires.

#### Statistical analyses

Statistical analyses were performed using IBM SPSS Statistics (Version 25). Descriptive statistics were used to summarize the sample characteristics. Corrected item-total correlation was used to evaluate how well items related to the instrument and to each other. Items with a corrected item-total correlation of less than 0.40 were deleted [19]. Subsequently, exploratory factor analyses were used to further screen and optimize the items, and KMO test (Kaiser-Meyer-Olkin (KMO) test) and Barlett’s spherical test were used to determine whether the scale data was suitable for EFA. When 0.6 and Bartlett’s sphericity test was statistically significant (*P*<0.05), it is considered that the data are suitable to proceed [20, 21]. The factor structure derived from the prior EFA was tested with confirmatory factor analysis (CFA). Within the scope of the CFA, the chi-square/degree of freedom (χ2/df < 5), goodness-of-fit index (GFI > 0.85), adjusted goodness-of-fit index (AGFI > 0.85), comparative fit index (CFI > 0.90), root mean square error of approximation (RMSEA < 0.08) and incremental fit index (IFI > 0.90) fit indices were used [22]. In the analyses of the content validity assessment of the scale items, the item-content validity index (I-CVI) and the scale-content validity index (S-CVI) were computed accordingly. Internal consistency reliability of the scale was examined using Cronbach’s α coefficient, with an acceptable cut-off value of ≥ 0.70 for the overall scale. The statistical significance level was accepted as *P* < 0.05.

#### Ethical approval

The Ethics Committee of Wuhan University School of Medicine issued an ethical license to the researchers with the ethical number (2020YF2001). All participants were informed about the study and volunteered to participate in the study. In addition, the researchers asked the subjects to sign an informed consent form to indicate their consent before recruitment. All methods were performed by relevant guidelines and regulations.

## Results

### Phase I: development of the items pool

In the qualitative interview, a total of 12 nurses were interviewed, all female, aged 25–49 years, with an average age of 35.17 ± 6.97 years; their years of experience in hospice care ranged from 2 to 11 years; 3 (25.0%) had junior titles, 6 (50.0%) had intermediate titles, and 3 (25.0%) had senior titles, the results of which are shown in Table 1. The results of the semi-structured interviews are detailed in *Supplementary Material 1*. The initial version scale was grouped into two dimensions and ten items, respectively: overall social environment (3 items), and organizational policy and culture (7 items). Detailed items for the initial version of the scale are shown in *Supplementary Material 2*.

**Table 1:** Demographic information of participants in semi-structured interviews

No	Gender	Age	Title	Education	Marital Status	Years in hospice care	Hospice wards	Attend hospice training	Specialized hospice nurse
N1	Female	31–35	Intermediate	Undergraduate	Married	3	Yes	Yes	Yes
N2	Female	36–40	Junior	Undergraduate	Married	2	Yes	Yes	Yes
N3	Female	41–45	Senior	Postgraduate	Married	4	No	Yes	Yes
N4	Female	26–30	Junior	Undergraduate	Married	2	No	No	No
N5	Female	31–35	Intermediate	Undergraduate	Married	3	No	Yes	Yes
N6	Female	46–50	Senior	Postgraduate	Married	10	No	Yes	Yes
N7	Female	21–25	Junior	Undergraduate	Married	3	Yes	No	No
N8	Female	26–30	Junior	Undergraduate	Married	5	No	No	No
N9	Female	36–40	Intermediate	Undergraduate	Married	5	Yes	Yes	Yes
N10	Female	31–35	Junior	Undergraduate	Married	3	Yes	Yes	Yes
N11	Female	31–35	Senior	Postgraduate	Married	5	Yes	Yes	Yes
N12	Female	41–45	Intermediate	Undergraduate	Married	11	No	Yes	Yes

### Phase II: selection of environment indicators through consensus process

The consensus process strictly followed the Delphi method. A total of 2 rounds of expert consultation were completed in this study. In each round, 16 experts were involved in the selection and optimization of scale dimensions and items. Among them, 15 were female and 1 was male, working in the fields of geriatrics, geriatric nursing, oncology nursing, and nursing education. Details of the experts are shown in the Table 2.

**Table 2:** Demographic of delphi’s participants (*N* = 16)

Variable	Category	Number	Proportion(%)
Gender	Male	1	6.3%
	Female	15	93.8%
Age	30–39	5	31.3%
	40–49	7	43.8%
	>50	4	25.0%
Educational level	Undergraduate	1	6.3%
	Master’s degree	9	56.3%
	Doctor	6	37.5%
Professional title	Intermediate	4	25.0%
	Auxiliary height	9	56.3%
	Positive height	3	18.8%
Working time	5–14 years	5	31.3%
	15–24 years	5	31.3%
	≥ 25 years	6	37.5%
Major field	Geriatric or oncology medicine	2	12.5%
	Geriatric or cancer care	8	50.0%
	Nursing education	6	37.5%
Postgraduate supervisor	Yes	7	43.8%
	No	9	56.3%
Mechanism	Hospital	10	62.5%
	Colleges and universities	6	37.5%

#### Round 1

The dimensions of the hospice care environment scale in the first round of expert consultation were 2. The coefficients of variation were 0.088 and 0.000, and the mean importance ratings were 3.875 and 4.000, with full score ratios of 87.5% and 100.0%, respectively, indicating a concentration of expert opinion on the importance of the two dimensions. The coefficients of variation of the 10 items of the scale ranged from 0.000 to 0.119, the mean importance scores ranged from 3.750 to 4.000, and the full score ratios ranged from 75.0 to 100.0%, and all indicators met the prespecified criteria, as shown in *Supplementary Material 3*. The first round of expert consultation form is available in *Supplementary Material 4.*

In the current round of expert consultation, experts suggested changing the dimensions to social and organizational environments. Three additional items were added: “Government administration established good hospice policy”, “Hospital/unit has good incentives for hospice work”, and “My department integrates multidisciplinary staffs (such as dietitians, social workers, volunteers, etc.) to provide hospice care services for patients”. In addition, some items were modified in expression. In this round of expert consultation, the I-CVI and S-CVI/Ave of the hospice care environment scale were both 1.000 and 1.000.

#### Round 2

In the second round of expert consultation, the coefficient of variation for each indicator ranged from 0.000 to 0.088, the mean importance score ranged from 3.875 to 4.000, and the full score ratio ranged from 87.5 to 100.0%, as shown in *Supplementary Material 3*. The second round of expert consultation form is available in *Supplementary Material 4.*

The experts pointed out that “multidisciplinary staff” should be listed in item 12. Therefore, we changed it to “The department integrates multidisciplinary staff (e.g., dietitians, social workers, volunteers, etc.) to provide hospice services to patients. There were no changes to the other items. In the current round of expert consultation, the I-CVI and S-CVI/Ave of the hospice care environment scale after adding 3 items were both 1.000.

After two rounds of expert consultation, the Hospice Care Environment Scale was developed, consisting of 2 dimensions and 13 items. A Likert 5-point scale was used (completely disagree = 1 point, disagree = 2 points, neutral = 3 points, agree = 4 points, and completely agree = 5 points).

### Phase III: psychometric evaluation of the hospice care environment scale

#### Participant characteristics

A total of 500 valid questionnaires were collected in this study. The valid questionnaires were randomly divided into two groups in a 1:1 ratio, Sample 1 (n_1_ = 250) for entry screening and Sample 2 (n_2_ = 250) for quality assessment of the formed scales. The descriptive statistics of the samples were used in Table 3, with the mean age of (32.36 ± 6.71) years for Sample 1, which ranged from 21 to 52 years, and (32.31 ± 6.91) years for Sample 2, which ranged from 20 to 54 years.

**Table 3:** Demographic and medical characteristics of participants (*N* = 500)

Variable	Category	Entry filtering (*n*_1_ = 250)		Scale quality evaluation(*n*_2_ = 250)	
		Number (*n*)	Constituent ratio(%)	Number (*n*)	Constituent ratio(%)
Gender	Male	2	0.8	2	0.8
	Female	248	99.2	248	99.2
Age	≤ 25	39	15.6	49	17.6
	26–30	66	26.4	54	21.6
	31–35	77	30.8	80	32.0
	36–40	34	13.6	36	14.4
	≥ 41	34	13.6	31	12.4
Marital status	Single	69	27.6	65	26.0
	Married	173	69.2	182	72.8
	Did not report	8	3.2	3	1.2
Nationality	Han nationality	245	98.0	241	96.4
	Ethnic minorities	5	2.0	9	3.6
Religion	Yes	9	3.6	11	4.4
	No	241	96.4	239	95.6
Educational level	Junior college	49	19.6	73	29.2
	Bachelor degree	201	80.4	177	70.8
Professional title	Primary	135	54.0	147	58.8
	Intermediate	95	38.0	91	36.4
	Advanced	20	8.0	12	4.8
Monthly income	<0.5	103	41.2	136	54.4
(Ten thousand)	0.5-1	143	57.2	111	44.4
	1-1.5	2	0.8	1	0.4
	>1.5	2	0.8	2	0.8
Post	General nurse	171	68.4	192	76.8
	Nursing team leader	8	3.2	8	3.2
	Specialist nurse	31	12.4	25	10.0
	Head nurse and above	40	16.0	25	10.0
Working hospital	General hospital	236	94.4	211	84.4
	Cancer hospital	14	5.6	39	15.6
Working time	≤ 5 years	84	33.6	83	33.2
	>5-≤10 years	75	30.0	80	32.0
	>10-≤15 years	51	20.4	45	18.0
	>15-≤20 years	16	6.4	21	8.4
	>20 years	24	9.6	21	8.4

#### Item analyses

The results of the critical ratio method showed that the scale items all had CR values > 3.0 and that the differences between the high and low subgroups were statistically significant for all entries (*P* < 0.0001). According to corrected item-total correlation analyses, since the correlation coefficients of the items were between 0.802 and 0.908, it was not considered necessary to remove items from the draft scale, and exploratory factor analysis was performed for the 13 items in the draft scale.

#### Exploratory factor analysis

**Fig. 1:**
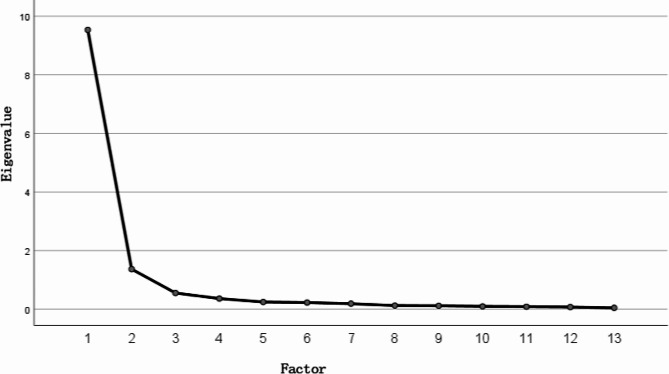
Parallel analyses for factor extraction(n_1_ = 250)

In the factor analyses, the Kaiser-Meyer-Olkin value was calculated as 0.927, while Bartlett’s test of sphericity statistic was calculated as χ2 = 4505.403,*P*<0.0001.These results revealed that the data set was large enough for factor analyses, could be considered homogeneous, and was suitable for factor analyses. According to the scree plot (Fig. 1), there is an inflection point at the 3rd factor, therefore, 2 factors were set to be extracted, and the principal component analyses method was chosen to extract them. The cumulative variance explained by the 2 factors was 83.856%, and after rotation using the direct oblique intersection method, the loadings on the factors to which each question item belonged were at a high level (0.619–1.003), and there were no large cross-loadings (< 0.4), and the factor structure clearly (Table 4). Factor 1 contains items 1–4, named “social environment”, and factor 2 contains items 5–13, named “organizational environment”.

**Fig. 2:**
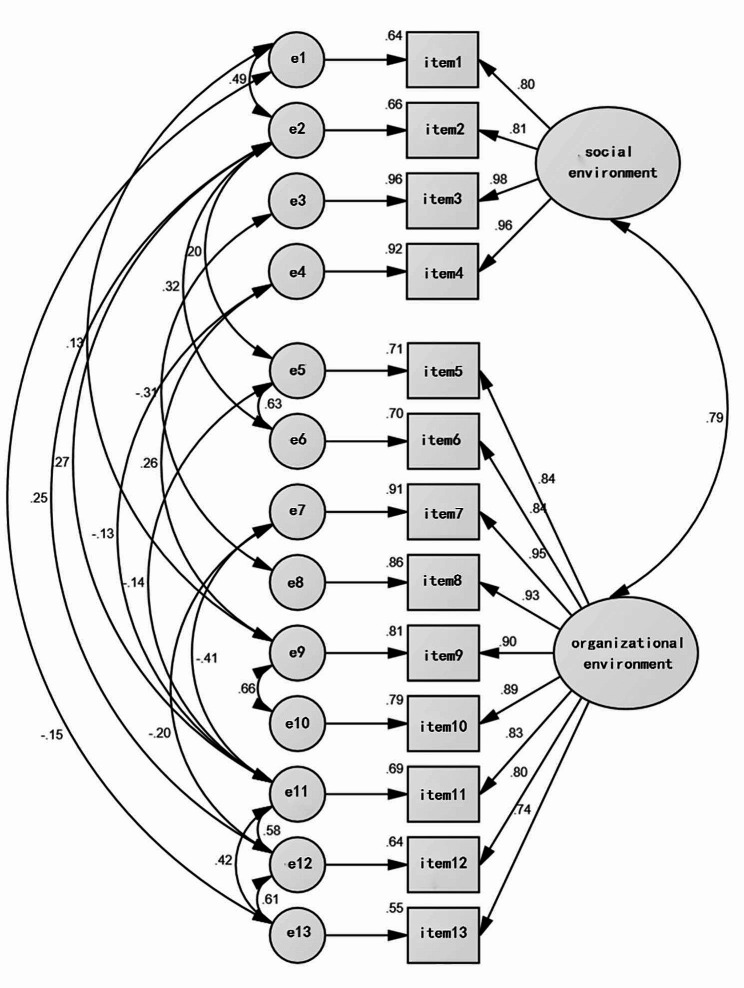
Standardized path chart of the hospice care environment scale

**Table 4:** Item characteristics and factor loadings of the hospice care environment scale

No.	Items	Factor and Factor Loadings	
		Factor1: Social Environment	Factor2: Organizational Environment
1	Government administration developed a comprehensive hospice policy.	0.782	0.173
2	Hospice care services are known and accepted by the public.	0.944	-0.006
3	Terminal patients recognize and receive hospice care services.	0.973	-0.028
4	Family members of terminal patients receive hospice care services.	0.924	0.025
5	My hospital/department has a hospice care management system.	0.103	0.858
6	My hospital/department has hospice care incentives.	0.110	0.848
7	Managers in my hospital/department recognize and actively promote the hospice care.	-0.105	1.003
8	Doctors in my department recognize and actively carry out hospice care.	0.005	0.948
9	Nurses in my department recognize and actively carry out hospice care.	-0.142	0.990
10	Medical staffs in my department can actively cooperate to complete the hospice care.	-0.066	0.957
11	Medical staffs in my department are sufficient to provide hospice care services.	0.179	0.762
12	My department integrates multidisciplinary staffs (such as dietitians, social workers, volunteers, etc.) to provide hospice care services for patients.	0.307	0.619
13	My department has good environmental facilities (such as single room, double room, aromatherapy oil, music, etc.) to provide hospice care services for patients.	0.257	0.641

#### Confirmatory factor analyses

The fit indices of the tested scale model were determined as χ2/df = 2.689, RMSEA = 0.082, CFI = 0.982, GFI = 0.932, AGFI = 0.866, IFI = 0.983. The structure of the scale was confirmed with 2 factors and 13 items (Fig. 2). In Table 5, the fit index values of the model tested for 13 items and the acceptable limits of these values are presented.

**Table 5:** Fit index values of the CFA of the hospice care environment scale

Index	Acceptable value	The scale
Chi-square/degree of freedom (χ2 /df)	<5	2.689
Goodness-of-fit index (GFI)	>0.85	0.932
Adjusted goodness-of-fit index (AGFI)	>0.85	0.866
Comparative fit index (CFI)	>0.90	0.982
Incremental fit index (IFI)	>0.90	0.983
Root means square error of approximation (RMSEA)	<0.08	0.082

#### Internal consistency reliability

The total Cronbach’s α coefficient of the hospice care environment scale was 0.970, among which the social environment dimension was 0.944 and the organizational environment dimension was 0.966, indicating that the internal consistency of the scale was good.

## Discussion

Evaluating hospice care environments is a critical step in promoting hospice care. Nurses are the major practitioners of hospice care. Therefore, we conducted development, validation, and reliability testing of the Hospice Care Environment Scale that is applicable to nurses. The Hospice Care Environment Scale contains 2 dimensions with 13 items and has been validated in nurses. The scale has good content and construct validity and is a reliable tool for assessing hospice care environment. To our knowledge, this is the first instrument specifically developed to assess hospice care environments and validated in China nurses.

In the Hospice Care Environment Scale, the environment is divided into social and organizational environments. The social environment is composed of the families to which we belong, the public in the communities in which we live, and the governments that establish policies [23]. The social environment dimension tends to assess whether the public, government, terminally ill patients, and families of terminally ill patients can agree and accept hospice care. Previous research has shown that the social environment affects the availability of hospice care. The government plays an important role in the hospice care environment. Government and policy makers may not sufficiently aware of the benefits and importance of hospice care in the world, thus stunting the development of hospice care [24]. Hospice care can reduces unnecessary hospitalization and use of health services, which reduces medical crowding and government expenditure [2]. In addition, the establishment of hospices does not place a monetary burden on the government because palliative care is not a high-cost expense compared to other professions [25]. Meanwhile, the public is equally unfamiliar with hospice care in the world. A cross-sectional survey of the Chinese public revealed that most Chinese knew little about hospice care, but most wanted to further learn more [24]. Patient and family who are reluctant to accept referrals for specialty hospice care may vary by culture, but the common thread of this hesitation is the association of hospice care with death [26]. In China and some countries, the traditional cultural concept of death and dying is a major obstacle to the development of hospice care. Only patients with cancer or those who are dying need hospice care [8]. In addition, there are some misconceptions about hospice care among the public. Hospice care is the absence of treatment for the patient and can accelerate the process of death. However, the evidence is now overwhelming that for patients with serious illness, access to hospice care is better than no access in all aspects, and early access is better than late [26–28].

The organizational environment dimension tends to assess whether hospital or institutional administrators, physicians, and nurses accept and promote the development of hospice care; and whether the department has sufficient medical professionals, multidisciplinary members, and equipment for hospice care. Lack of financial cost and guidelines are the most important reasons that hinder the development of hospice care [6]. Although the World Health Organization issued guidelines for hospice care managers in 2016 [29] and the Chinese Health Commission issued the Hospice Care Practice Guidelines in 2017 [1], there is still variation in whether they can be implemented specifically to each healthcare facility. In 2020, a study suggested that only a quarter of Chinese healthcare professionals were knowledgeable about hospice care [6]. Healthcare providers in China may not be familiar with hospice care or may be reluctant to propose hospice care [10]. Training programs for health care providers on the availability and appropriate use of hospice care are needed. Therefore, it is appropriate that we developed the scale in terms of the social and organizational environment dimensions.

This study strictly followed the principles and process recommended by Robert F. DeWillis [13]. To ensure the scientific validity of the scale development, the Delphi method was used to screen and optimize the dimensions and entries of the Hospice Care Environment Scale in this study [18]. A total of 16 experts with solid professional backgrounds and academic experience were invited to conduct the correspondence consultation. The response rates of the two rounds of consultation were 88.9% and 100.0%, which were higher than the recommended 70% [18], indicating the high participation and enthusiasm of the experts. The authority coefficients of experts in the two rounds of consultation were 0.853 and 0.869, representing the authority and credibility of Delphi consultation. The overall Cronbach’s α coefficient for the Hospice Care Environment Scale was 0.970, and the Cronbach’s α coefficients for the 2 dimensions were 0.952 and 0.969, both of which were greater than the criterion of 0.8, indicating that the two scales had good internal consistency and good overall reliability. Validity can be divided into content validity and structural validity. The I-CVI of the Hospice Care Environment Scale ranged from 0.917 to 1.000, and the S-CVI was 1, both of which were higher than the recommended criteria for content validity of the scale. The factor loadings of all items of the Hospice Care Environment Scale were greater than 0.5, and the convergent and discriminant validity met the desired criteria. In conclusion, the scale is a reliable instrument to assess the hospice care environment.

Shaping the hospice care environment to facilitate the desired outcomes requires valid and reliable measurements to assess the practice environment before, during, and after changes are implemented. After nurses can evaluate the hospice care environment by using this scale, it will be clear what the current state of the environment is at the site. Policymakers can use the results to target improvements to the weak aspects of the hospice care environment and make improvements to sites with poor hospice environments in order to promote hospice care accessibility and health equity.

Limitations also exist in the current study. First, the scale was developed based on oncology nurses in China, and its applicability in other countries and regions needs further validation. Furthermore, concurrent validity and retest reliability were not conducted in this study. However, currently evidence suggests that this scale is a reliable tool for assessing the hospice care environment. Finally, the scale developed in this study addresses only nurses’ perceptions of the hospice care environment and ignores the perspectives of multidisciplinary staff, such as dietitians, social workers, and volunteers. Future research should explore the views of other professional groups on the environment to fully assess the importance of the hospice care environment.

## Conclusion

The process of this study followed strictly the procedure of scale development. The hospice care environment scale we developed had good reliability and validity and was tested in Chinese nurses. This study provides a new perspective for assessing hospice care development in terms of socio-cultural and organizational dimensions. The scale may provide a reliable reference for assessing hospice care environment in China and other countries.

### Electronic supplementary material

Below is the link to the electronic supplementary material.

Supplementary Material 1

Supplementary Material 2

## Data Availability

No datasets were generated or analysed during the current study.
