# Supplementary material for: Development, validation and reliability testing of the hospice care environment scale
Source: BMC Palliat Care. 2024 May 28;23:135. doi: 10.1186/s12904-024-01450-2 (PMC11131208; doi:10.1186/s12904-024-01450-2)
Supplement: Supplementary file 1 — Supplementary Material 1 [file 12904_2024_1450_MOESM1_ESM.docx]

**Hospice Care Environment Scale (Chinese Version)**

**安宁疗护环境量表**

指导语：请您根据自己的认知或工作经验进行判断，在合适的选项内打“✓”。

|  | 完全不同意 | 不同意 | 中立 | 同意 | 完全同意 |
| --- | --- | --- | --- | --- | --- |
| 1政府管理部门制定了良好的安宁疗护政策。 |  |  |  |  |  |
| 2社会大众知晓并认可安宁疗护服务。 |  |  |  |  |  |
| 3终末期患者认可并接受安宁疗护服务。 |  |  |  |  |  |
| 4终末期患者家属认可并接受安宁疗护服务。 |  |  |  |  |  |
| 5我所在医院/科室有良好的安宁疗护工作的管理制度。 |  |  |  |  |  |
| 6我所在医院/科室有良好的安宁疗护工作的激励措施。 |  |  |  |  |  |
| 7我所在医院/科室管理人员认可并积极推动安宁疗护工作。 |  |  |  |  |  |
| 8我所在科室医生认可并积极开展安宁疗护工作。 |  |  |  |  |  |
| 9我所在科室护士认可并积极开展安宁疗护工作。 |  |  |  |  |  |
| 10我所在科室医护人员能积极合作完成安宁疗护工作。 |  |  |  |  |  |
| 11我所在科室有充足的医护人员提供安宁疗护服务。 |  |  |  |  |  |
| 12我所在科室整合多学科人员（如营养师、社工、志愿者等）为患者提供安宁疗护服务。 |  |  |  |  |  |
| 13我所在科室有良好的环境设施（如单间、双间、香薰油、音乐等）为患者提供安宁疗护服务。 |  |  |  |  |  |

**Hospice Care Environment Scale (English Translation Version)**

**Hospice Care Environment Scale**

Note: Please use your own judgement based on your own perception or work experience by ticking "✓" in the appropriate box.

|  | Completely disagree | Disagree | Impartiality | Agree | Completely agree |
| --- | --- | --- | --- | --- | --- |
| 1.Government administration developed a comprehensive hospice policy. |  |  |  |  |  |
| 2.Hospice care services are known and accepted by the public. |  |  |  |  |  |
| 3.Terminal patients recognize and receive hospice care services. |  |  |  |  |  |
| 4.Family members of terminal patients receive hospice care services. |  |  |  |  |  |
| 5.My hospital/department has a hospice care management system. |  |  |  |  |  |
| 6.My hospital/department has hospice care incentives. |  |  |  |  |  |
| 7.Managers in my hospital/department recognize and actively promote the hospice care. |  |  |  |  |  |
| 8.Doctors in my department recognize and actively carry out hospice care. |  |  |  |  |  |
| 9.Nurses in my department recognize and actively carry out hospice care. |  |  |  |  |  |
| 10.Medical staffs in my department can actively cooperate to complete the hospice care. |  |  |  |  |  |
| 11.Medical staffs in my department are sufficient to provide hospice care services. |  |  |  |  |  |
| 12.My department integrates multidisciplinary staffs (such as dietitians, social workers, volunteers, etc.) to provide hospice care services for patients. |  |  |  |  |  |
| 13.My department has good environmental facilities (such as single room, double room, aromatherapy oil, music, etc.) to provide hospice care services for patients. |  |  |  |  |  |
