# Supplementary material for: Development, validation and reliability testing of the hospice care environment scale
Source: BMC Palliat Care. 2024 May 28;23:135. doi: 10.1186/s12904-024-01450-2 (PMC11131208; doi:10.1186/s12904-024-01450-2)
Supplement: Supplementary file 2 — Supplementary Material 2 [file 12904_2024_1450_MOESM2_ESM.doc]

**Supplementary Material 1**

**Extraction of scale items from semi-structured interview (examples**)

| **Dimension** | **Item** | | **Interview information (examples)** |
| --- | --- | --- | --- |
| Overall Social Environment | | 1.Hospice care services are known and accepted by the community. | N11: It is important for all the public to have the idea to accept this event, that death is not a particularly terrible or taboo thing, but an integral part of life. If society as a whole can change this concept, perhaps everyone will accept this event more calmly, rather than particularly resistant or even aggressive, and if the public can change that perception and then change their behavior, then it is possible to carry out better. |
|  |  | 2. Terminal patients recognize and accept hospice services. | N5: There is a patient, I served with him for almost two months, he once said to me, Mr. Chen, don't come into my ward anymore, for the patient, I think it's hard to talk to the nurse about the meaning of life or some rational issues if you say you can't receive hospice care. |
|  |  | 3. Family members of terminal ill patients recognize and accept hospice services. | N6: The family has to accept the fact, sometimes when the patient is only just coming in before the diagnosis is confirmed, the family is going to be devastated and he thinks there is no hope for me with this disease when the hospice nurse comes. |
| Organizational policies and culture | | 4. The hospital has developed a comprehensive system to manage and promote hospice care. | N9: In the department, I think the environment of the workplace and patient wards, as well as the improvement of the reward mechanism for work performance, can make people more motivated. |
|  |  | 5. Hospital and department managers actively promote the development of hospice care. | N1: Leadership awareness is very important, you have a policy, the leader does not understand anything, and you have no one to tell him, the leader does not pay attention to how he goes to support this thing, so we need to say repeatedly. |
|  |  | 6. The department physician recognizes and actively carries out hospice care. | N12: Doctors need to have the concept to do some other aspects of support, such as the support of the treatment plan, maybe we will be able to go more smoothly, or we will progress more deeply. |
|  |  | 7. The department has adequate medical staff to provide hospice care. | N3: In hospice care, it's not just a nurse, it requires the support of a whole team of rehabilitators, pharmacologists, nutritionists, and then nurses and social workers. |
|  |  | 8. The department has ample support equipment to allow me to spend more time providing hospice care to my patients. | N15: I think the first is our hospital environment, if there is a single room to put these patients in a single room, I think it is better, and then is actually like some patients believe in Buddhism or believe in Christianity, we can give some cultural support, and then like some patients may more like singing or something else, we can give him some music or aromatherapy. |

**Supplementary Material 2**

**Hospice Care Environment Scale (Items pool**)

| **Dimension** | **Item** |
| --- | --- |
|  | 1.Hospice care services are known and accepted by the public. |
|  | 2.Terminal patients recognize and receive hospice care services. |
|  | 3.Family members of terminal patients receive hospice care services. |
| Organizational Policy and Culture | 4. The hospital has developed a comprehensive system to manage and promote hospice care. |
|  | 5. Hospital and department managers actively promote the development of hospice care. |
|  | 6. The department physician recognizes and actively carries out hospice care. |
|  | 7. The nurses in the department recognize and actively support the development of hospice care. |
|  | 8. The medical and nursing staff of the department can actively cooperate to complete hospice care. |
|  | 9. The department has adequate medical staff to provide hospice care. |
|  | 10. The department has adequate support equipment to allow me to spend more time providing hospice care to patients. |

**Supplementary Material 3**

Hospice Environment Scale dimensions and items importance rating results (Round 1)

| Dimension | Item | CV | Average score | Full Score Ratio |
| --- | --- | --- | --- | --- |
| Overall Social Environment |  | 0.088 | 3.875 | 87.5% |
|  | 1.Hospice care services are known and accepted by the community. | 0.119 | 3.750 | 75.0% |
|  | 2.Terminal patients recognize and receive hospice care services. | 0.000 | 4.000 | 100.0% |
|  | 3.Family members of terminal patients receive hospice care services. | 0.000 | 4.000 | 100.0% |
| Organizational Policy and Culture |  | 0.000 | 4.000 | 100.0% |
|  | 4. The hospital has developed a comprehensive system to manage and promote hospice care. | 0.000 | 4.000 | 100.0% |
|  | 5. Hospital and department managers actively promote the development of hospice care. | 0.000 | 4.000 | 100.0% |
|  | 6. The department physician recognizes and actively carries out hospice care. | 0.000 | 4.000 | 100.0% |
|  | 7. The nurses in the department recognize and actively support the development of hospice care. | 0.000 | 4.000 | 100.0% |
|  | 8. The medical and nursing staff of the department can actively  cooperate to complete hospice care. | 0.088 | 3.875 | 87.5% |
|  | 9. The department has adequate medical staff to provide hospice care. | 0.000 | 4.000 | 100.0% |
|  | 10. The department has adequate support equipment to allow me to spend more time providing hospice care to patients. | 0.000 | 4.000 | 100.0% |

Hospice Environment Scale dimensions and items importance rating results (Round 2)

| Dimension | Item | CV | Average score | Full Score Ratio |
| --- | --- | --- | --- | --- |
| Social Environment |  | 0.088 | 3.875 | 87.5% |
|  | 1. Government administration established good hospice policy. | 0.000 | 4.000 | 100.0% |
|  | 2.Hospice care services are known and accepted by the public. | 0.000 | 4.000 | 100.0% |
|  | 3.Terminal patients recognize and receive hospice care services. | 0.000 | 4.000 | 100.0% |
|  | 4.Family members of terminal patients receive hospice care services. | 0.000 | 4.000 | 100.0% |
| Organizational Environment |  | 0.000 | 4.000 | 100.0% |
|  | 5.My hospital/department has a hospice care management system. | 0.000 | 4.000 | 100.0% |
|  | 6.My hospital/department has hospice care incentives. | 0.000 | 4.000 | 100.0% |
|  | 7.Managers in my hospital/department recognize and actively promote the hospice care. | 0.000 | 4.000 | 100.0% |
|  | 8.Doctors in my department recognize and actively carry out hospice care. | 0.088 | 3.875 | 87.5% |
|  | 9.Nurses in my department recognize and actively carry out hospice care. | 0.000 | 4.000 | 100.0% |
|  | 10.Medical staffs in my department can actively cooperate to complete the hospice care. | 0.000 | 4.000 | 100.0% |
|  | 11.Medical staffs in my department are sufficient to provide hospice care services. | 0.000 | 4.000 | 100.0% |
|  | 12.My department integrates multidisciplinary staffs (such as dietitians, social workers, volunteers, etc.) to provide hospice care services for patients. | 0.000 | 4.000 | 100.0% |
|  | 13.My department has good environmental facilities (such as single room, double room, aromatherapy oil, music, etc.) to provide hospice care services for patients. | 0.000 | 4.000 | 100.0% |

**Supplementary Material 4**

**Correspondence Form for Dimension of Hospice Care Environment Scale (**Round 1)

| Dimension | Definition | Correlation | | | | Importance | | | | Delete, modify and comment |
| --- | --- | --- | --- | --- | --- | --- | --- | --- | --- | --- |
|  |  | Uncorrelated  1 | Weak correlated  2 | Comparative Correlated  3 | Correlated  4 | Unimportant  1 | Not very important  2 | Comparative important  3 | Very important  4 |  |
| Overall social environment | It refers to the national policy and guidelines on hospice care and the public’s understanding of palliative care. |  | | | |  | | | |  |
| Organization policy and culture | It refers to the policy, system and culture of hospice care in hospitals and departments, and the understanding, recognition and implementation of hospice care by medical colleagues. |  | | | |  | | | |  |
| Additional items |  |  | | | |  | | | |  |
|  |  |  |  |  |  |  |  |  |  |  |

**Correspondence Form for Items of Hospice Care Environment Scale (**Round 1)

| Dimension | Definition | Correlation | | | | Importance | | | | Delete, modify and comment |
| --- | --- | --- | --- | --- | --- | --- | --- | --- | --- | --- |
|  |  | Uncorrelated  1 | Weak correlated  2 | Comparative Correlated  3 | Correlated  4 | Unimportant  1 | Not very important  2 | Comparative important  3 | Very important  4 |  |
| Overall social environment | 1. Hospice care is recognized and accepted by the public. |  | | | |  | | | |  |
|  | 2. Terminal patients are recognized and receive hospice care services |  | | | |  | | | |  |
|  | 3. Family members of terminally ill patients receive hospice care services. |  | | | |  | | | |  |
| Organizational policy and culture | 4. The hospital has developed a comprehensive system to manage and promote hospice care. |  | | | |  | | | |  |
|  | 5. Hospital and department managers actively promote the development of hospice care. |  | | | |  | | | |  |
|  | 6. The department physician recognizes and actively carries out hospice care. |  | | | |  | | | |  |
|  | 7. The nurses in the department recognize and actively support the development of hospice care. |  | | | |  | | | |  |
|  | 8. The medical staff in the department can cooperate actively to complete the hospice care. |  | | | |  | | | |  |
|  | 9. The department has adequate medical staff to provide hospice care. |  | | | |  | | | |  |
|  | 10. The department has adequate support equipment to allow me to spend more time providing hospice care to patients. |  | | | |  | | | |  |
| Additional items |  |  | | | |  | | | |  |
|  |  |  |  |  |  |  |  |  |  |  |

**Correspondence Form for Items of Hospice Care Environment Scale (Round 2)**

| Dimension | Definition | Correlation | | | | Importance | | | | Delete, modify and comment |
| --- | --- | --- | --- | --- | --- | --- | --- | --- | --- | --- |
|  |  | Uncorrelated  1 | Weak correlated  2 | Comparative Correlated  3 | Correlated  4 | Unimportant  1 | Not very important  2 | Comparative important  3 | Very important  4 |  |
| Social environment | It refers to the national policies and guidelines on hospice care and the public’s understanding of hospice care. |  | | | |  | | | |  |
| Organizational environment | It refers to the hospice policy, system and culture of hospitals and departments, and the understanding, recognition and implementation of hospice care by medical colleagues. |  | | | |  | | | |  |
| Additional items |  |  | | | |  | | | |  |
|  |  |  |  |  |  |  |  |  |  |  |

**Correspondence Form for Items of Hospice Care Environment Scale (Round 2)**

| Dimension | Definition | Correlation | | | | Importance | | | | Delete, modify and comment |
| --- | --- | --- | --- | --- | --- | --- | --- | --- | --- | --- |
|  |  | Uncorrelated  1 | Weak correlated  2 | Comparative Correlated  3 | Correlated  4 | Unimportant  1 | Not very important  2 | Comparative important  3 | Very important  4 |  |
| Social environment | 1.Government administration established good hospice policy. |  | | | |  | | | |  |
|  | 2. Hospice care services are known and accepted by the public. |  | | | |  | | | |  |
|  | 3.Terminal patients recognize and receive hospice care services. |  | | | |  | | | |  |
|  | 4.Family members of terminal patients receive hospice care services. |  | | | |  | | | |  |
| Organizational environment | 5.My hospital/department has a hospice care management system. |  | | | |  | | | |  |
|  | 6.My hospital/department has hospice care incentives. |  | | | |  | | | |  |
|  | 7.Managers in my hospital/department recognize and actively promote the hospice care. |  | | | |  | | | |  |
|  | 8.Doctors in my department recognize and actively carry out hospice care. |  | | | |  | | | |  |
|  | 9.Nurses in my department recognize and actively carry out hospice care. |  | | | |  | | | |  |
|  | 10.Medical staffs in my department can actively cooperate to complete the hospice care. |  | | | |  | | | |  |
|  | 11.Medical staffs in my department are sufficient to provide hospice care services. |  | | | |  | | | |  |
|  | 12.My department integrates multidisciplinary staffs (such as dietitians, social workers, volunteers, etc.) to provide hospice care services for patients. |  | | | |  | | | |  |
|  | 13.My department has good environmental facilities (such as single room, double room, aromatherapy oil, music, etc.) to provide hospice care services for patients. |  | | | |  | | | |  |
| Additional items |  |  | | | |  | | | |  |
|  |  |  |  |  |  |  |  |  |  |  |
